# Acute Effects of Ethanol on Hippocampal Spatial Representation and Offline Reactivation

**DOI:** 10.3389/fncel.2020.571175

**Published:** 2020-11-05

**Authors:** Kosaku Miyake, Saichiro Yagi, Yuki Aoki, Yu Shikano, Yuji Ikegaya, Takuya Sasaki

**Affiliations:** ^1^Graduate School of Pharmaceutical Sciences, University of Tokyo, Tokyo, Japan; ^2^Center for Information and Neural Networks, Suita, Japan; ^3^Precursory Research for Embryonic Science and Technology, Japan Science and Technology Agency, Saitama, Japan

**Keywords:** ethanol, hippocampus, place cell, synchronization, reactivation

## Abstract

Acute alcohol exposure impairs hippocampus-dependent spatial memory. However, there is little evidence for the effects of ethanol on the spike patterns of hippocampal cell populations. Here, we examined how the spatial firing patterns of place cells, neurons that encode specific locations, were altered in rats that were intraperitoneally injected with 1.5 g/kg ethanol. Ethanol administration partly reduced or abolished place-selective spiking of a subset of place cells during running periods in a spatial task, whereas a subset of place fields newly emerged, suggesting a partial reorganization of hippocampal spatial maps by ethanol. On the other hand, ethanol administration did not significantly alter the frequency of hippocampal sharp-wave ripple (SWRs) and synchronous spike patterns during resting periods, suggesting that offline memory consolidation and retrieval mechanisms underpinned by hippocampal neuronal synchronization are not strongly affected by ethanol. These results indicate that acute ethanol intake mainly affects the encoding of external information but has little impact on internal memory processing.

## Introduction

Acute ethanol overconsumption causes marked impairment in a variety of brain functions, including cognition, learning, and memory (Acheson et al., 1998), which have been well replicated in laboratory rodent animals. The deleterious effects of ethanol are mainly mediated by the disruption of hippocampal functions (Ryabinin, 1998; White et al., 2000; Kutlu and Gould, 2016). At the microscopic level, the effects of ethanol on numerous molecular targets in neurons and synapses within the hippocampus have been identified, including a reduction in glutamate release (Martin and Swartzwelder, 1992; Shimizu et al., 1998), suppression of *N*-methyl-D-aspartate (NMDA) receptor-mediated ion currents (Lovinger et al., 1989), and potentiation of gamma-aminobutyric acid A (GABA_A_) receptor-mediated ion currents (Kumar et al., 2009), all of which cause alternations of both excitatory and inhibitory synaptic transmission (Lovinger, 1997). Moreover, acute ethanol acts on diverse receptors and ion channels in many subcortical regions innervating the hippocampus (Abrahao et al., 2017). *In vivo* electrophysiological recordings have revealed that ethanol administration suppresses cellular activity in the medial septum (Givens, 1996), increases the spike rates of dopamine neurons in the ventral tegmental area (VTA; Gessa et al., 1985; Burkhardt and Adermark, 2014), and alters the firing patterns of distinct types of neurons in the central amygdala (Herman and Roberto, 2016). All this evidence indicates that unlike those of compounds that target a single molecule, the mechanisms underlying ethanol-induced changes in hippocampal functions are highly complex, and ethanol-induced hippocampus-dependent behavior is associated with a myriad combination of changes in neuronal activity in the intrahippocampal circuit and extrahippocampal regions (Abrahao et al., 2017). To elucidate the precise mechanisms underlying ethanol-related behavior, further studies are required to examine how integrated ethanol-sensitive mechanisms lead to alterations in neuronal spike dynamics in the hippocampus.

Over the last few decades, several works have addressed this issue by utilizing extracellular recordings of hippocampal neuronal spikes in freely moving rodents (Grupp and Perlanski, 1979; Ludvig et al., 1995; Tokunaga et al., 2003). These studies have consistently demonstrated that acute ethanol administration inhibits the spike rates of hippocampal neurons. Moreover, recordings during spatial tasks have shown that ethanol administration reduces the place-selective firing of hippocampal place cells (Matthews et al., 1996; White and Best, 2000), pyramidal neurons that specifically discharge in restricted locations of an environment (O’Keefe and Dostrovsky, 1971). However, in these early studies, spike data were recorded from a small number of cells (mostly fewer than 10) using single electrodes, and only simple measures such as changes in spike rate and spatial selectivity were analyzed.

Data recording techniques have been prominently advanced by the use of tetrode arrays with 10 of channels (Wilson and McNaughton, 1994; Aoki et al., 2019). Also, our knowledge has increased regarding the detailed dynamics of hippocampal place cell populations, such as the remapping of spatial maps in response to changes in the external environment (Leutgeb et al., 2004) and sharp-wave ripple (SWR)-associated offline memory reactivation (Lee and Wilson, 2002; Girardeau et al., 2009). With these recently developed recording techniques and insights, we systematically analyzed how the spike patterns of larger numbers of hippocampal cells (166 cells) are affected by acute ethanol. The results implicate the neurophysiological mechanisms underlying ethanol-induced changes in cognition and memory.

## Materials and Methods

### Animal Ethics

This study was performed in strict accordance with the recommendations of the NIH Guide for the Care and Use of Laboratory Animals. All animals were handled according to the approval of the experimental animal ethics committee at the University of Tokyo (approval number: P29-11).

### Subjects

Seven male Long Evans rats (3–6 months old) with a preoperative weight of 381–433 g were used in this study. The animals were individually housed and maintained on a 12-h light/dark schedule with lights off at 7:00 AM. All animals were purchased from SLC (Shizuoka, Japan). Following at least 1 week of adaptation to the laboratory environment, the rats were reduced to 85% of their *ad libitum* weight through limited daily feeding for behavioral tasks. Water was readily available.

### Surgical Procedures

The rats were anesthetized with isoflurane gas (1.5–2.5%). A craniotomy with a diameter of ~2 mm was performed using a high-speed drill, and the dura was surgically removed. Two stainless-steel screws were implanted in the bone above the cerebellum to serve as the ground and reference electrodes. An electrode assembly that consisted of 16 independently movable tetrodes (Okada et al., 2017; Yagi et al., 2018; Aoki et al., 2019), which was created using a 3D printer (Form 2, Formlabs^1^), was stereotaxically implanted above the right hippocampus (3.8 mm posterior and 2.7 mm lateral to the Bregma). The electrodes were constructed from a 17-μm-wide polyimide-coated platinum-iridium (90/10%) wire (California Fine Wire), and the electrode tips were plated with platinum to lower the electrode impedance to 150–300 kΩ at 1 kHz. The recording device was secured to the skull using stainless-steel screws and dental cement. The electrode bundle tip was lowered to the cortical surface, and the electrodes were inserted 1.0 mm into the brain at the end of the surgery. Following the surgery, each rat was individually housed in a transparent Plexiglass cage with free access to water and food for at least 3 days, followed by food deprivation to 85% of its original body weight.

### U-Track Task

**Figure 1:**
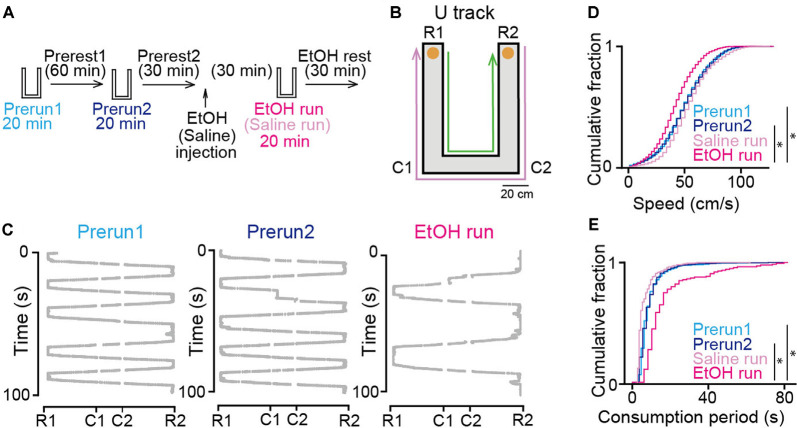
Effects of ethanol on animal behavior on the U-track. **(A)** Experimental timeline. Each run session was 20 min, and inter-run intervals (rest) were >30 min. Ethanol or saline was injected into the rats after the Prerest2 session. The EtOH and saline run sessions commenced 30 min after ethanol administration. **(B)** An overview of the U-track. Notable locations are labeled C1, C2, R1, and R2. Reward locations are indicated by orange circles. The magenta and green arrows show the trajectories from R2 to R1 and R1 to R2, respectively. **(C)** Representative 100-s running trajectories in individual run sessions in a rat. **(D)** Cumulative distributions of instantaneous running speed on the track. *P < 0.05, Mann–Whitney *U* test followed by Bonferroni correction. **(E)** Cumulative distributions of stay duration at consummatory areas per lap on the track. *P < 0.05, Mann–Whitney *U* test followed by Bonferroni correction.

All behavioral experiments occurred in the dark. The rats were trained daily on a U-shaped track task for at least 9 days (Figure 1A). On one training day, the rat was trained to run back and forth on a U-shaped track consisting of two 100 × 9 cm^2^ and one 50 × 9 cm^2^ alleyway (with small sides rising 5.0 cm above the surface of the arm, 90 cm elevated from the floor) to obtain a constant ~0.2 ml of chocolate milk reward placed at the end of the track during a 20 min session. This training was repeated daily for 20–60 min. On some training days, the training was performed with the recording headstage and cable attached to the animals so that the animals could become familiar with the recording condition. To monitor the rat’s moment-to-moment position, a red LED was attached to the microdrive, and the LED signal position was tracked at 25 Hz using a video camera located on the ceiling and sampled by a laptop computer. The rats were maintained in a rest box (30 × 30 cm^2^) outside the field before and after the task.

### Electrophysiological Recordings

The rats were connected to the recording equipment *via* a Cereplex M (Blackrock), a digitally programmable amplifier placed close to the rat’s head. The output of the headstage was conducted *via* a lightweight multiwire tether and a commutator to a Cerebus recording system (Blackrock). Electrode turning was performed while the rat was resting on a pot placed on a pedestal. Over at least 2 weeks after surgery, the electrode tips were slowly advanced 20–250 μm per day for up to 30 days until spiking cells were encountered in the CA1 layer of the hippocampus, which were identified based on local field potential (LFP) signals and single-unit spike patterns. The tetrodes were settled into the cell layer to obtain stable recordings over several days. After confirming that stable, well-separated unit activity could be identified in the hippocampus and that the animal reached the threshold performance, electrophysiological data were collected during the tasks for at least 20 min. LFP recordings were sampled at 2 kHz and low-pass filtered at 500 Hz. The unit activity was amplified and bandpass filtered from 500 Hz to 6 kHz. Spike waveforms above a trigger threshold (80 μV) were time-stamped and recorded at 30 kHz for 1.6 ms.

### Histological Analysis

The rats received an overdose of urethane and were intracardially perfused with 4% paraformaldehyde in phosphate-buffered saline (pH 7.4) and decapitated. To aid in electrode track reconstruction, the electrodes were not withdrawn from the brain for at least 3 h after perfusion. Following dissection, the brains were fixed overnight in 4% PFA and subsequently equilibrated with 30% sucrose in saline. Frozen coronal sections (50 μm) were cut using a microtome, and serial sections were mounted and processed for cresyl violet staining. The slices were subsequently coverslipped with Permount. The positions of all tetrodes were confirmed by identifying the corresponding electrode tracks in the histological tissue.

### Spike Sorting

**Figure 2:**
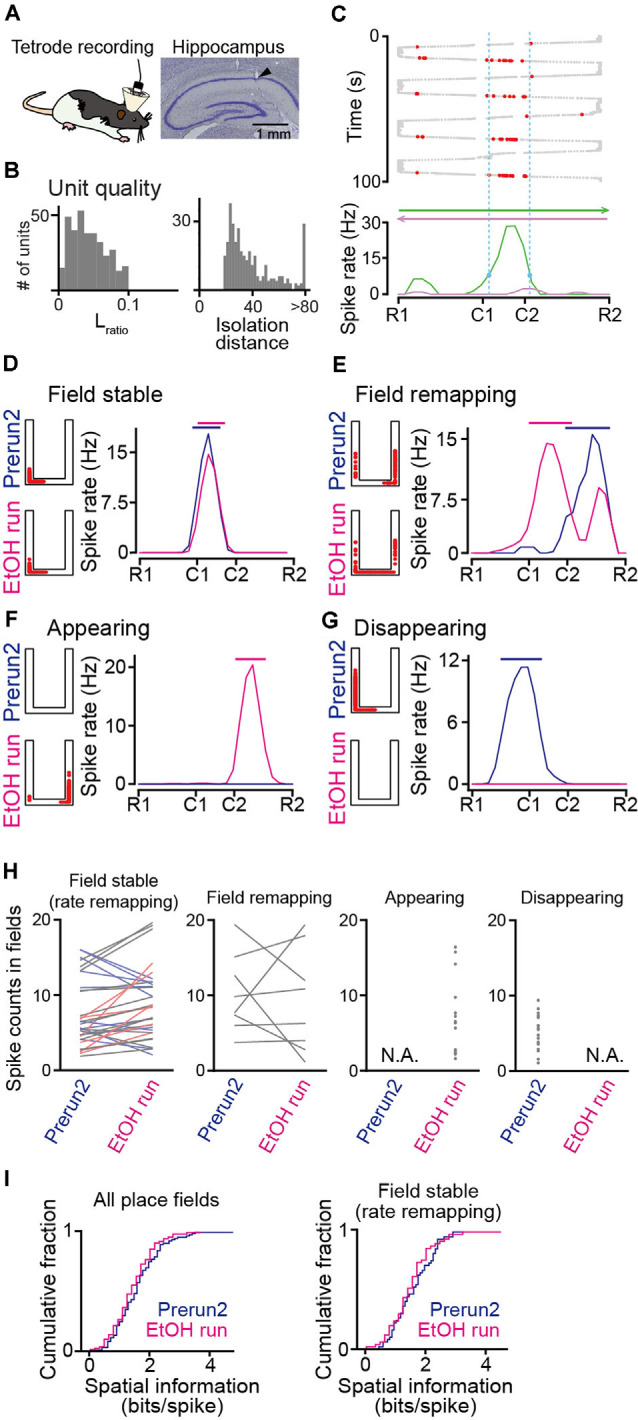
Recording of place cell firing in ethanol-injected rats. (**A**; Left) Tetrode recordings were performed from the dorsal hippocampal CA1 area in freely moving rats. (Right) Histological confirmation of a recording site in the CA1 cell layer in a cresyl-stained section. The arrowhead indicates the track of the electrode. **(B)** The distributions of *L*_ratio_ and isolation distance for individual neuronal units, identified by multiunit spike sorting. **(C)** The spatial firing of a representative place cell. (Top) Spike locations are indicated by the red dots superimposed on the animal’s trajectory (thick and thin gray dots). (Bottom) Spatial firing-rate distributions constructed from the firing locations above separately analyzed for each running direction. The range of the place field is indicated by the dashed vertical cyan lines. **(D–G)** Representative place cells showing typical firing characteristics before (Prerun2) and after (EtOH run) administration of ethanol (**D**, field stable; **E**, field remapping; **F**, appearing; and **G**, disappearing). In each graph, the left panel shows the spike locations as red dots superimposed on the maze structure, and the right panel shows the spatial firing-rate distributions (blue, Prerun2; magenta, EtOH run). The bars above indicate the areas of the place fields. **(H)** Ethanol-induced changes in spike counts per lap within place fields, separately plotted for field stable with rate remapping, field remapping, appearing, and disappearing fields (*n* = 50, 8, 19, and 43 fields). Each line and each dot represent an individual place cell. Stable place fields were further classified into no rate remapping (gray, *n* = 25 fields) or rate remapping (increase: light red, *n* = 6 fields; decrease: light blue, *n* = 19 fields). Sessions with no place fields identified were represented as N.A. **(I)** Cumulative distributions of spatial information of all place fields identified (left) and stable fields (right). *P* > 0.05, Kolmogorov–Smirnov test.

Spike sorting was performed offline using the graphical cluster-cutting software MClust. Clustering was performed manually in 2D projections of the multidimensional parameter space [i.e., comparisons between waveform energies and first principal component (PC1) of the energy normalized waveform, each measured from the four channels of each tetrode]. The cluster quality was measured by computing *L*_ratio_ and Isolation Distance (Schmitzer-Torbert et al., 2005). A cluster was considered as a cell when the *L*_ratio_ was less than 0.10 and the Isolation Distance was more than 19 (Figure 2B). Cells with an average spike rate of less than 3 Hz and waveforms longer than 200 μs were considered putative excitatory cells and included in the analysis.

### Analysis of Spatial Firing

The track was divided into two parts: (i) the consummatory areas, the two regions within 20 cm from either track end; and (ii) the running area, the U-track minus the consummatory areas. Consummatory periods were defined as the periods during which the animals were in the consummatory area. An instantaneous speed for each frame was calculated based on the total distance traveled within a period of four frames (~160 ms) before and after the focused frame. A lap was judged as a “running lap” if the animal’s instantaneous running speed exceeded 30 cm/s within the running area after departing from a previous consummatory area without running backwards until reaching the opposite consummatory area. Laps that did not meet these criteria were those that were judged to include decelerating, stopping, or returning behaviors and were excluded from further analyses.

For each cell, an average spatial spike rate distribution with a bin size of 10 cm was computed from all running laps in one direction. Here, as the number of running laps in the EtOH session was fewer than that in the prerest sessions (Figure 1D), spatial spike rate distributions in each animal were computed from the first *N* laps in all sessions, given that the number of laps in the EtOH session was *N* in a direction. All spike-rate distributions were smoothed by a one-dimensional convolution with a Gaussian kernel with a standard deviation of one pixel (10 cm). A place cell was defined based on the two following criteria. The first criterion was that the maximum spike rate in the average spatial spike-rate distribution exceeded >3 Hz. The second criterion was that the maximum spike rate exceeded two standard deviations (SDs) above the mean, where the SD and the mean were computed from the series of spike rates except the maximum spike rate in the distribution. In cells that met these two criteria, the range of spatial spiking, termed the place field, was defined by finding the bin with the maximum spiking rate in the spatial-spiking rate distribution and then iteratively extending the field to any adjacent bins that had spiking rates of >30% of the maximum rate. If a cell has multiple place fields, a place field with the maximum spike rate was selected as its place field. Cells that met all of these criteria were classified as place cells. Under these criteria, some cells had one place field with one of two directions (unidirectional place field), whereas the other cells had two place fields with both directions (bidirectional place fields). In the following analyses, bidirectional place fields from a single place cell were counted as two place-fields and separately analyzed. For each running lap, spike counts and spike rates were calculated over the periods when the rats passed the place fields in one direction.

For each place-field identified in a session, spatial information (SI), the information density measured in bits per spike, was computed as follows:

(1) $$\text{SI}=\sum_{i=1}^{N}p_{i}\frac{r_{i}}{r}\log_{2}\frac{r_{i}}{r}$$

where *i* is the index of the bins (10 cm) of the place field, *p_i_* is the probability of the animal being at location *i*, *r_i_* is the average firing rate of the neuron when the animal is at location *i*, and *r* is the total average firing rate.

### SWR Detection and Synchronous Events

To detect SWRs from hippocampal LFP signals, the electrode including the largest number of HPC pyramidal cells identified in the spike sorting process was used. The LFP signal was bandpass filtered at 150–250 Hz, and the root-mean-square power was calculated in the ripple band with a bin size of 24 ms. The threshold for SWR detection was set to three SDs above the mean. Synchronous events were detected when: (1) ≥30% of hippocampal cells active were simultaneously activated in a 200-ms time window that was preceded by >200 ms of silence and (2) the running speed of the animals was less than 5 cm/s.

### Cofiring

To measure the degree to which a given cell pair exhibited synchronous spikes, termed cofiring (O’Neill et al., 2008), the numbers of spikes were counted in consecutive 200-ms windows in each of the two cells, creating *N*-dimensional vectors *x* and *y*, where *N* is the total number of windows. Pearson’s correlation coefficients were computed between the two vectors as follows:

(2) $$\text{Cofiring}=\frac{\sum_{i=1}^{N}(x_{i}-\bar{x})(y_{i}-\bar{y})}{\sqrt{\sum_{i=1}^{N}{\left(x_{i}-\bar{x}\right)}^{2}}\sqrt{\sum_{i=1}^{N}{\left(y_{i}-\bar{y}\right)}^{2}}}$$

this analysis was applied to all possible cell pairs in which the total number of time windows (200 ms) with synchronous spikes was more than 50.

### Explained Variance

Based on the cofiring maps constructed above, an explained variance (EV) across a given neighboring rest session (rest1 and rest2) was computed as follows (Kudrimoti et al., 1999; Giri et al., 2019):

(3) $$\text{EV}={\left(\frac{R_{\text{run,rest2}}-R_{\text{run,rest1}}\times R_{\text{rest1,rest2}}}{\sqrt{\left(1-R_{\text{run,rest1}}^{2}\right)\left(1-R_{\text{rest1,rest2}}^{2}\right)}}\right)}^{2}$$

where *R*_run,rest1_ is a correlation of cofiring maps of all neurons between run (between rest1 and rest2) and rest1 sessions. To compute reversed EV (REV) as a control measure, cofiring maps were reversed between the rest1 and rest2 sessions.

### Statistics

The significance of the correlation between two variables was evaluated by computing the Pearson correlation coefficients. Comparisons of two-sample data were analyzed *via* the Mann–Whitney *U* test. A comparison of ratio distributions of place field types between two groups were assessed using the Chi-square test. The null hypothesis was rejected at the *P* < 0.05 level unless otherwise specified. All measurements are reported as the means ± standard errors of the mean (SEMs) and were analyzed using Python and MATLAB.

## Results

### Ethanol-Administered Rats in a U-Track Task

The experimental timeline of this study is shown in Figure 1A. The rats performed a running task in which they ran back and forth on a U-shaped track with a chocolate milk reward placed at both ends of the track for 20 min (Figure 1B), termed run sessions. To confirm the stability of the animal’s behavior and neuronal firing patterns in the task, two run-sessions (Prerun1 and Prerun2) were tested with an intersession interval of 60 min in a rest box (Prerest1). Following a 30-min rest period (Prerest2), the rats were injected with saline or 1.5 g/kg ethanol (i.p.), a dose comparable to those generally consumed by humans. After the next 30-min rest period, the rats were tested in an identical run session, termed the Saline and EtOH run session. Finally, the rats were again placed back in the rest box and allowed to rest for 30 min (EtOH rest). Representative trajectories of a rat from the three run-sessions are presented in Figure 1C. On average, a 20-min run session before (Prerun1 and Prerun2) and after (EtOH run) ethanol injection included 57 ± 28 and 23 ± 13 running laps per session, respectively. Overall, the distributions of instantaneous running speed did not significantly differ between the Prerun1 and Prerun2 sessions (Figure 1D; *U* = 18, *P* = 0.47, Mann–Whitney *U* test followed by Bonferroni correction). Also, no significant difference in duration in which the rats stayed in the consummatory areas was found between the two run-sessions (Figure 1E; *U* = 44, *P* = 0.34, Mann–Whitney *U* test followed by Bonferroni correction). These results confirm the stability of the animal’s behavioral patterns across run sessions before ethanol injection. In saline-injected rats, running speeds and stay durations in the consummatory areas did not significantly differ from those before the injection (Figure 1D; Prerun1 vs. Saline run, *U* = 5, *P* = 0.24; Prerun2 vs. Saline run, *U* = 5, *P* = 0.24; Figure 1E; Prerun1 vs. Saline run, *U* = 6, *P* = 0.33; Prerun2 vs. Saline run, *U* = 6, *P* = 0.33, Mann–Whitney *U* test followed by Bonferroni correction), confirming that aversive experience of the injection itself did not affect animal’s running behavior. On the other hand, the animals’ running speeds and stay durations in the consummatory areas after ethanol injection was significantly decreased and increased, respectively (Figure 1D; Prerun1 vs. EtOH run, *U* = 6, *P* = 0.030; Prerun2 vs. EtOH run, *U* = 2, *P* = 0.007; Figure 1E; Prerun1 vs. EtOH run, *U* = 12, *P* = 0.030; Prerun2 vs. EtOH run, *U* = 9, *P* = 0.010, Mann–Whitney *U* test followed by Bonferroni correction). These results demonstrate that systemic administration of ethanol in our experimental condition partially reduces locomotor behavior but did not diminish their motivation to perform the task. These behavioral changes are likely explained by several mechanisms outside the hippocampus, including ataxia mediated by the motor system and cerebellum, decreased attention mediated by widespread neocortical and subcortical regions, and altered cerebral blood flow through changes in systemic blood pressure.

### Hippocampal Place Cells in Ethanol-Administered Rats

To monitor hippocampal neuronal spikes during the task, we implanted 16 movable tetrodes into the dorsal hippocampal CA1 region in the rats (Figure 2A). In total, 166 and 185 hippocampal cell units were isolated from six rats (ethanol-injected group) and two rats (saline-injected group) performing the task, respectively (Figure 2B). Figure 2C shows the spatial firing patterns of a representative place cell with clear place-selective spikes in one direction (green) but not in the opposite direction (magenta). In all rats tested, of the 137 place cells identified, 66 (48.2%) cells had unidirectional place fields depending on the moving direction, whereas 71 (51.8%) cells had bidirectional place fields independent of the movement direction. Here, the bidirectional place fields from a single cell were counted as two place-fields, resulting in a total of 213 place fields analyzed. For each place cell, spatial firing patterns were compared between the Prerun2 and EtOH run sessions. As the total number of laps in the EtOH session was fewer than that before ethanol injection, the number of laps to compute spatial spike-rate distributions in each animal was set to the number of laps in the EtOH session. Figure 2D shows a place cell that initially existed in the Prerun2 session and showed no pronounced changes in its preferred location in the EtOH run session. Here, such place fields that shifted their center with a distance of less than 30 cm across sessions were classified as a “field stable” type (*n* = 50 fields; Figure 2H, leftmost panel). Within the stable place fields, some neurons changed their spike rate by more than 50% across sessions, which were further classified as “rate remapping” type (Figure 2H, leftmost panel, colored lines). Figure 2E shows a place field that shifted to a new location at a distance of more than 30 cm in the EtOH run session and was classified as a “field remapping” type. Changes in spike counts within place fields classified as the field remapping type are shown in the second panel in Figure 2H (*n* = 8 fields). Figure 2F shows a place field that was newly generated in the EtOH run session and was classified as an “appearing” type (*n* = 19 fields; Figure 2H, third panel). In contrast, Figure 2G shows a place field that was identified in the Prerun2 session but was absent in the EtOH run session, which was classified as a “disappearing” type (*n* = 43 fields; Figure 2H, rightmost). For place fields identified in these sessions, spatial information density was computed (Figure 2I). No significant differences in spatial information of all place fields were found between the Prerun2 and EtOH sessions (Figure 2I, left; *D*_max_ = 0.12, *P* = 0.49, Kolmogorov–Smirnov test). This result was similar when the analysis was restricted to only stable place fields (Figure 2I, right; *D*_max_ = 0.14, *P* = 0.62, Kolmogorov–Smirnov test). These results suggest that ethanol did not prominently affect the strength of spatial tuning of individual place fields.

**Figure 3:**
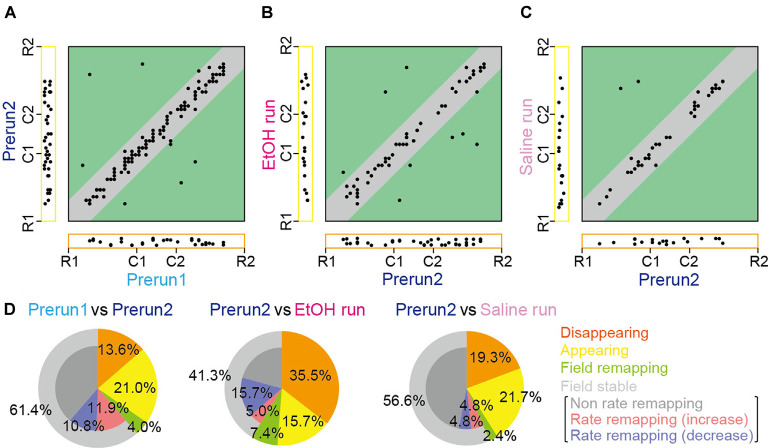
Changes in place cell firing patterns in ethanol-treated rats. **(A)** For each place field, the position of the place field center in the Prerun1 session was plotted against that in the Prerun2 session (*n* = 176 fields). Each dot represents an individual field. The Left and bottom areas represent fields that exhibited no spatial firing in one session but exhibited spatial firing in the other session, classified as appearing and disappearing types, respectively. Types of changes in spatial firing patterns are shown in the colored areas as follows: orange, disappearing; yellow, appearing; green, remapping; and gray, stable. **(B)** Same as **(A)** but plotted for firing patterns in the EtOH run session against those in the Prerun2 session (*n* = 121 fields). **(C)** Same as **(A)** but plotted for firing patterns in the Saline run session against those in the Prerun2 session (*n* = 83 fields). **(D)** The proportions of place field types, analyzed from **(A–C)**.

However, overall proportions of place fields were prominently changed by ethanol. The positions of all place fields observed across sessions are summarized in Figures 3A–C. Out of the 176 fields identified in the Prerun2 session in saline-injected and ethanol-injected rats, the majority (61.4%) of place fields stably remained at an identical location to that of the Prerun1 session, confirming the stability of fields across run sessions (Figure 3D). After ethanol administration, this percentage was significantly reduced to 41.3% (50/121 fields; *χ*^2^ = 11.1, *P* < 0.001, chi-square test), whereas such difference was not observed in the saline-injected rats (*χ*^2^ = 0.53, *P* = 0.47, chi-square test). Furthermore, in these stable place fields, 38.0% of the neurons decreased their in-field spike rates [as represented by rate remapping (decrease)] in the ethanol-injected rats, which was significantly higher than the ratios in the no drug-injected rats (Prerun1 and Prerun2; *χ*^2^ = 6.46, *P* = 0.022, chi-square test followed by Bonferroni correction) and saline-injected rats (*χ*^2^ = 12.8, *P* < 0.001, chi-square test followed by Bonferroni correction), suggesting their reduced spatial encoding by ethanol. Also, disappearing fields in the ethanol-injected rats accounted for 35.5% of all fields initially identified in the Prerun2 session (Prerun2 and EtOH run), which was significantly higher than the ratios in the no drug-injected rats (*χ*^2^ = 20.1, *P* < 0.001, chi-square test followed by Bonferroni correction) and saline-injected rats (*χ*^2^ = 6.52, *P* = 0.011, chi-square test followed by Bonferroni correction). These results suggest that the overall spatial representations by place cell populations are partly reduced by ethanol primarily due to significant reductions in in-field firing rates (decreased rate remapping) and significant increases in the fractions of disappearing place fields. The proportions of place fields showing field remapping or appearing in the EtOH run session were not significantly different from those in the saline run session (field remapping, 7.4%, *χ*^2^ = 1.07, *P* = 0.30, chi-square test; appearing, 15.7%; *χ*^2^ = 1.25, *P* = 0.26, chi-square).

### Hippocampal SWRs in Ethanol-Administered Rats

**Figure 4:**
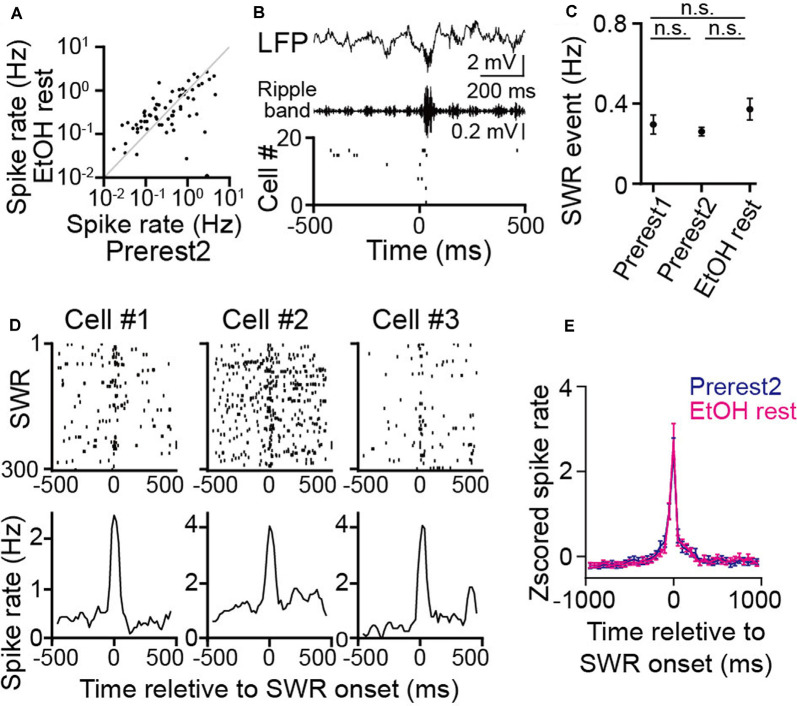
No pronounced changes in hippocampal sharp-wave ripple (SWR) events during rest in ethanol-treated rats. **(A)** Comparison of average spike rates in individual neurons before (Prerest2) and after (EtOH rest) ethanol administration (*n* = 166 neurons). Each dot represents an individual cell. (**B**; From top to bottom) An local field potential (LFP) trace, the corresponding ripple band (150–250 Hz)-filtered LFP trace, and a raster plot of the spike patterns of 20 hippocampal pyramidal cells. In the raster plot, each dot represents an individual spike. An SWR event and the corresponding synchronized spikes occur at 0 ms. **(C)** The frequency of SWR events in each rest session (*n* = 6 rats). *P* > 0.05, paired *t*-test followed by Bonferroni correction. **(D)** Three representative neurons are shown. (Top) Raster plots are aligned to SWR onset in an individual cell. (Bottom) The corresponding instantaneous spike rates are relative to the SWR onset. **(E)** Comparison of *Z-scored* SWR-triggered averages of spike rate changes (*n* = 166 neurons) between the Prerest2 (blue) and EtOH rest (magenta) sessions. *Z-scores* were calculated for the spike rate through each rest. n.s.: not significant.

While animals are resting/sleeping during postexperience periods, hippocampal cells are continuously active, potentially reflecting memory consolidation. Next, we analyzed the effects of ethanol on such “offline” spike patterns during the rest sessions. No significant differences in the overall spike rates were found between the Prerest2 and EtOH rest sessions (Figure 4A; *t*_(165)_ = 0.96, *P* = 0.34, paired *t*-test). Next, we asked whether ethanol affects synchronized spikes in neuronal populations, which are considered crucial for neuronal plasticity and memory consolidation. First, we detected hippocampal SWRs from the LFP signals (Figure 4B), which typically represent the synchronous firing of hippocampal pyramidal cells (Csicsvari et al., 2000; Buzsáki, 2015). No significant differences in the frequency of the SWRs were found among the Prerest1, Prerest2, and EtOH rest sessions (Figure 4C; *P* > 0.05 across sessions, paired *t*-test followed by Bonferroni correction). To examine whether these SWRs were functionally different across the synchronizing neurons, we computed the SWR-triggered spike rates for individual place cells that had place fields during the Prerun2 or EtOH run periods (Figure 4D, three representative neurons). Overall, there were no significant differences in the SWR-triggered spike rates between the Prerest2 and EtOH rest sessions (Figure 4E; *t*_(165)_ = 1.54, *P* = 0.13, paired *t*-test). These results demonstrate that ethanol had no pronounced effects on the generation of hippocampal SWRs or the magnitude of SWR-related spike synchrony.

### Cofiring of Hippocampal Neurons in Ethanol-Treated Rats

**Figure 5:**
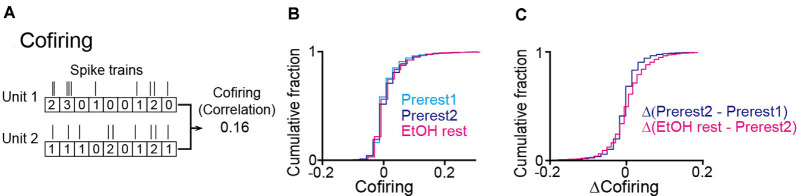
No pronounced changes in the cofiring of neuron pairs during rest periods in ethanol-treated rats. **(A)** An example of a cofiring analysis between a pair of neurons. Cofiring was computed as a correlation coefficient of spike counts (bin = 200 ms) of two neurons. **(B)** Cumulative distributions of cofiring of all neuron pairs (*n* = 3,893 neuron pairs from six rats). **(C)** Cumulative distributions of differences in cofiring between the two rest sessions.

To further examine in detail whether the synchronized spikes of specific neuron groups were altered by ethanol, we analyzed the degree of neuronal cofiring as the correlation coefficient of spike timing between a neuron pair (O’Neill et al., 2008). For each rat, we constructed a cofiring map for each session by computing the cofiring from all possible neuron pairs (Figure 5A). In the pooled data from all animals, no significant differences in cofiring were found across the three sessions (Figure 5B; Prerest1 vs. Prerest2: *U* = 12, *P* = 0.19; Prerest2 vs. EtOH rest: *U* = 14, *P* = 0.29, Mann–Whitney *U* test followed by Bonferroni correction), demonstrating that positive neuronal cofiring appeared to the same extent before and after ethanol administration. To examine whether the contents of the cofiring maps (i.e., patterns of neuron pairs with pronounced cofiring) were changed by ethanol, we computed the differences in cofiring for individual neuron pairs between the Prerest2 and EtOH sessions, termed Δ(EtOH–Prerest2). As a control, we computed the cofiring differences between the Prerest1 and Prerest2 sessions, termed Δ(Prerest2–Prerest1), representing the baseline changes in cofiring between the two rest sessions without ethanol. Overall, we found no significant differences between Δ(EtOH–Prerest2) and Δ(Prerest2–Prerest1; Figure 5C; *U* = 12, *P* = 0.19, Mann–Whitney *U* test).

**Figure 6:**
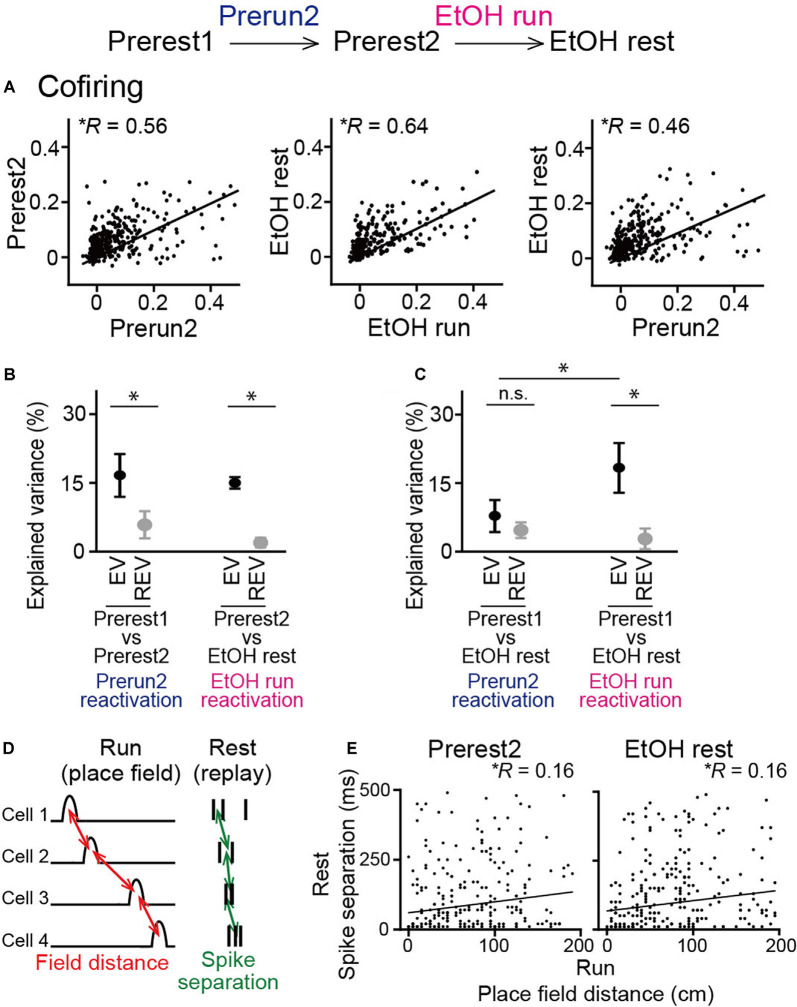
No pronounced changes in cofiring of place cell pairs during rest periods in ethanol-treated rats. **(A)** Cofiring of place cell pairs between a run session and the subsequent rest session (left, Prerun2 vs. Prerest2; middle, EtOH run vs. EtOH rest; right, Prerun2 vs. EtOH rest). Each dot represents each cell pair. All graphs showed significant positive correlations (*P* < 0.001). Only place cells identified in each run session were included for this analysis (*n* = 384, 475, and 384 neuron pairs from six rats). **(B)** Explained variance (EV) across groups of sessions (*n* = 6 rats). Two rest sessions and one run session used for the analysis were described below. In both groups of sessions, EV was significantly higher than the reversed EV (REV). *P < 0.05, paired *t*-test. **(C)** Same as B but for the Prerest1 session vs. the EtOH session. *P < 0.05, paired *t*-test followed by Bonferroni correction. **(D)** Schematic illustration of sequential reactivation analysis. For each place cell pair, a place field distance between the two cells during the previous run session (red, field distance) and an absolute average difference in spike timing between the two cells during a rest session (green, spike separation) were computed. **(E)** Relationship between place field distance and spike separation for all place cell pairs (Prerest2: *n* = 339 neuron pairs; EtOH rest: *n* = 334 neuron pairs). Each dot represents each cell pair. Both graphs showed significant positive correlations (*P* < 0.01). n.s.: not significant.

The analysis above examined synchronous spike patterns from all neurons recorded. We next focused specifically on place cells, which was considered to represent memory reactivation of spatial maps constructed during the run sessions. For all place cell pairs, cofiring in two rest sessions was plotted against that in the prior run sessions (Figure 6A). In both groups of sessions, significant positive correlations were observed when plotted against the Prerun2 and EtOH run sessions, respectively (left, Prerest2: *n* = 384 neuron pairs, *R* = 0.56, *P* < 0.001; middle, EtOH rest: 475 neuron pairs, *R* = 0.64, *P* < 0.001), suggesting reactivation patterns of place cell ensembles in subsequent rest periods are structured even when ethanol was administrated. We then plotted cofiring patterns in the EtOH rest session against those in the Prerun2 session (Figure 6A, right). In this case, the correlation was reduced to 0.46, while it was still a significant positive correlation (*n* = 384 neuron pairs, *P* < 0.001). This result was consistent with the observations that ethanol partially changed spatial encoding patterns of place cells (as described in Figures 2, 3) and suggests that place cell ensemble patterns that are more specific to the EtOH run session are more strongly reactivated in the EtOH rest session. To test this idea, based on these cofiring of all place cells across sessions, we calculated EV in each animal to examine the degree to which neuronal cofiring patterns during a run session are included in the subsequent rest session, compared with a prior rest session (Kudrimoti et al., 1999; Giri et al., 2019; Figure 6B). To test whether EV could be explained by a chance level, reversed explained variance (REV) was calculated as a control measure by reversing the two rest periods. Average EV computed across the Prerest1 and Prerest2 sessions based on the Prerun2 session was significantly higher than the corresponding REV (*t*_(5)_ = 3.55, *P* = 0.040, paired *t*-test), again verifying that neuronal pairs that are activated during a run session are more preferentially reactivated in the subsequent rest period without ethanol. Consistent with the cofiring patterns in Figure 6A, the same significant result was observed from EV computed across the Prerest2 and EtOH rest sessions based on the EtOH run session (*t*_(5)_ = 7.20, *P* = 0.0060, paired *t*-test). These results demonstrate that reactivation of place cell ensembles occurs in an ethanol-injected condition, suggesting that ethanol did not prominently disrupt memory reactivation mechanisms. To further test their detailed reactivation patterns, we computed EV across the Prerest1 and EtOH rest sessions based on individual run sessions (Figure 6C). The EtOH rest session, compared with the Prerest1 session, did not show significantly higher EV than the corresponding REV when computed based on cofiring patterns in the Prerun2 session (*t*_(5)_ = 1.15, *P* = 0.30, paired *t*-test followed by Bonferroni correction), meaning that these reactivation patterns occur at chance level. On the other hand, similar to the results in Figure 6B, it showed significantly higher EV than REV when computed based on the EtOH run session (*t*_(5)_ = 4.23, *P* = 0.0082, paired *t*-test followed by Bonferroni correction). Consistently, the EV based on the EtOH run session was significantly higher than that on the Prerun2 session (*t*_(5)_ = 3.29, *P* = 0.021, paired *t*-test followed by Bonferroni correction). These results demonstrate that place cell spike patterns that become unique after, but not before, ethanol administration is significantly recruited in reactivated patterns during the subsequent rest periods in the ethanol-administrated condition.

Finally, we analyzed sequential reactivation patterns of place cells, a potential substrate of replays of place cell ensembles within short time (hundreds of milliseconds) windows during subsequent rest periods, which are considered crucial for offline memory consolidation (Lee and Wilson, 2002; Girardeau et al., 2009). Here, due to limited numbers of recorded neurons, we employed pairwise comparisons between a pair of neurons to detect spike patterns that potentially represent ordered replays of behaviorally relevant sequences (Singer and Frank, 2009; Carr et al., 2011; Suh et al., 2013). For a given place cell pair, the distance between the two place-fields during the run sessions and the average time differences in their spike timing during synchronization (termed spike separation) was computed (Figure 6D). If the two variables were positively correlated in the pooled data from all neuron pairs, it would suggest that the place cell populations represent organized sequential reactivation patterns during the rest periods. In both the Prerest2 and EtOH rest sessions, we found significant positive correlations between place field distance and spike separation (Figure 6E; Prerest2: *n* = 339 neuron pairs, *R* = 0.16, *P* = 0.003; EtOH rest: *n* = 334 neuron pairs, *R* = 0.16, *P* = 0.004). These results demonstrate that the reactivation patterns of hippocampal place cells are not strongly affected by ethanol.

## Discussion

It is well known that the acute intake of ethanol causes pronounced impairment in recognition and memory both in humans and rodents. The hippocampus has been implicated as a brain region involved in these ethanol effects (Ryabinin, 1998; White et al., 2000; Kutlu and Gould, 2016). In this study, by utilizing multiunit recording techniques, we systematically examined how ethanol affects the more detailed dynamics of place cell ensembles, such as remapping of spatial maps during running periods, SWRs, and synchronized reactivations during resting periods.

The heterogeneous effects of ethanol among cells, including place and nonplace cells, might be explained by different levels of ethanol sensitivity in the receptors and synapses in cell subpopulations. For example, the mechanisms of ethanol action include the reduced release of glutamate (Martin and Swartzwelder, 1992; Shimizu et al., 1998), reduced NMDA receptor-mediated currents (Lovinger et al., 1989), and increased GABA_A_ receptor-mediated currents (Kumar et al., 2009), which result in inhibitory effects on neuronal activity. However, when these mechanisms act on inhibitory interneurons, disinhibition would lead to increased excitation of specific hippocampal cells and might even create new place fields, as observed in our study. Moreover, the transfer of neuromodulators to the hippocampus is a possible mechanism involved in the ethanol effect. Ethanol has been shown to reduce cholinergic signals through the inhibition of cellular activity in the medial septum (Givens, 1996) and enhance dopaminergic signals through the excitation of cellular activity in the VTA (Gessa et al., 1985; Burkhardt and Adermark, 2014). Heterogeneous functional projections from the extrahippocampal regions to individual hippocampal cells may account for the differences in ethanol sensitivity of individual place cells. Taken together, the integration of these intrahippocampal and extrahippocampal factors possibly leads to the complex diversity of ethanol-induced changes in the spatial representations in individual place cells (Abrahao et al., 2017).

Previous studies have demonstrated that ethanol decreases the overall frequencies of the place-selective firing of hippocampal place cells (Matthews et al., 1996; White and Best, 2000), suggesting reduced spatial representations. Our results demonstrated that nearly half of the place cell population retained their place fields at identical locations, suggesting that spatial cognition could be partly maintained even in the presence of ethanol. However, firing rates within such stable place fields were significantly decreased by ethanol, consistent with the previous studies. These detailed changes may serve as possible neuronal substrates for ethanol-induced errors of spatial recognition and memory.

Hippocampal SWR events and synchronous neuronal spikes during rest/sleep periods have been thought to play a pivotal role in memory consolidation and retrieval (Csicsvari et al., 2000; Buzsáki, 2015). Considering our observation that ethanol administration did not significantly change such organized hippocampal activity patterns, ethanol had no pronounced effects on offline memory processing. Together with our insights that spatial representation is reduced by ethanol, we suggest that ethanol mainly suppresses the memory acquisition phase in response to external environments rather than the memory consolidation phase after an experience. Taken together, our results implicate a neurophysiological mechanism underlying ethanol-induced impairments in cognition and memory. Further studies are necessary to elucidate how ethanol-induced neuronal activity in the hippocampus influences neuronal populations in downstream extrahippocampal brain regions and results in the expression of ethanol-sensitive behavioral actions.

## Data Availability Statement

The raw data supporting the conclusions of this article will be made available by the authors, without undue reservation.

## Ethics Statement

The animal study was reviewed and approved by the experimental animal ethics committee at the University of Tokyo (approval number: P29-11).

## Author Contributions

KM and TS designed the work. KM, SY, YA, and YS acquired electrophysiological data and performed the analysis. YI supervised the project. KM prepared all figures. TS wrote the main manuscript text. Authors reviewed the main manuscript text. All authors contributed to the article and approved the submitted version.

## Conflict of Interest

The authors declare that the research was conducted in the absence of any commercial or financial relationships that could be construed as a potential conflict of interest.

## Footnotes

^1^The stl files are available at: https://data.mendeley.com/datasets/pmgddv4c4v/2

## References

[B1] Abrahao K. P. Salinas A. G. Lovinger D. M. (2017). Alcohol and the brain: neuronal molecular targets, synapses, and circuits. Neuron 96, 1223–1238. 10.1016/j.neuron.2017.10.032 29268093 PMC6566861

[B2] Acheson S. K. Stein R. M. Swartzwelder H. S. (1998). Impairment of semantic and figural memory by acute ethanol: age-dependent effects. Alcohol. Clin. Exp. Res. 22, 1437–1442. 10.1111/j.1530-0277.1998.tb03932.x 9802525

[B3] Aoki Y. Igata H. Ikegaya Y. Sasaki T. (2019). The integration of goal-directed signals onto spatial maps of hippocampal place cells. Cell Rep. 27, 1516.e5–1527.e5. 10.1016/j.celrep.2019.04.002 31042477

[B4] Burkhardt J. M. Adermark L. (2014). Locus of onset and subpopulation specificity of *in vivo* ethanol effect in the reciprocal ventral tegmental area-nucleus accumbens circuit. Neurochem. Int. 76, 122–130. 10.1016/j.neuint.2014.07.006 25058792

[B5] Buzsáki G. (2015). Hippocampal sharp wave-ripple: a cognitive biomarker for episodic memory and planning. Hippocampus 25, 1073–1188. 10.1002/hipo.22488 26135716 PMC4648295

[B6] Carr M. F. Jadhav S. P. Frank L. M. (2011). Hippocampal replay in the awake state: a potential substrate for memory consolidation and retrieval. Nat. Neurosci. 14, 147–153. 10.1038/nn.2732 21270783 PMC3215304

[B7] Csicsvari J. Hirase H. Mamiya A. Buzsáki G. (2000). Ensemble patterns of hippocampal CA3-CA1 neurons during sharp wave-associated population events. Neuron 28, 585–594. 10.1016/s0896-6273(00)00135-5 11144366

[B8] Gessa G. L. Muntoni F. Collu M. Vargiu L. Mereu G. (1985). Low doses of ethanol activate dopaminergic neurons in the ventral tegmental area. Brain Res. 348, 201–203. 10.1016/0006-8993(85)90381-6 2998561

[B9] Girardeau G. Benchenane K. Wiener S. I. Buzsaki G. Zugaro M. B. (2009). Selective suppression of hippocampal ripples impairs spatial memory. Nat. Neurosci. 12, 1222–1223. 10.1038/nn.2384 19749750

[B10] Giri B. Miyawaki H. Mizuseki K. Cheng S. Diba K. (2019). Hippocampal reactivation extends for several hours following novel experience. J. Neurosci. 39, 866–875. 10.1523/JNEUROSCI.1950-18.2018 30530857 PMC6382986

[B11] Givens B. (1996). Behavioral correlates of single units in the medial septal area: the effect of ethanol. Neuroscience 71, 417–427. 10.1016/0306-4522(95)00443-2 9053797

[B12] Grupp L. A. Perlanski E. (1979). Ethanol-induced changes in the spontaneous activity of single units in the hippocampus of the awake rat: a dose-response study. Neuropharmacology 18, 63–70. 10.1016/0028-3908(79)90010-8 418956

[B13] Herman M. A. Roberto M. (2016). Cell-type-specific tonic GABA signaling in the rat central amygdala is selectively altered by acute and chronic ethanol. Addict. Biol. 21, 72–86. 10.1111/adb.12181 25170988 PMC4345144

[B14] Kudrimoti H. S. Barnes C. A. McNaughton B. L. (1999). Reactivation of hippocampal cell assemblies: effects of behavioral state, experience, and EEG dynamics. J. Neurosci. 19, 4090–4101. 10.1523/JNEUROSCI.19-10-04090.1999 10234037 PMC6782694

[B15] Kumar S. Porcu P. Werner D. F. Matthews D. B. Diaz-Granados J. L. Helfand R. S.. (2009). The role of GABA_A_ receptors in the acute and chronic effects of ethanol: a decade of progress. Psychopharmacology 205, 529–564. 10.1007/s00213-009-1562-z 19455309 PMC2814770

[B16] Kutlu M. G. Gould T. J. (2016). Effects of drugs of abuse on hippocampal plasticity and hippocampus-dependent learning and memory: contributions to development and maintenance of addiction. Learn. Mem. 23, 515–533. 10.1101/lm.042192.116 27634143 PMC5026208

[B17] Lee A. K. Wilson M. A. (2002). Memory of sequential experience in the hippocampus during slow wave sleep. Neuron 36, 1183–1194. 10.1016/s0896-6273(02)01096-6 12495631

[B18] Leutgeb S. Leutgeb J. K. Treves A. Moser M. B. Moser E. I. (2004). Distinct ensemble codes in hippocampal areas CA3 and CA1. Science 305, 1295–1298. 10.1126/science.1100265 15272123

[B19] Lovinger D. M. (1997). Alcohols and neurotransmitter gated ion channels: past, present and future. Naunyn Schmiedebergs Arch. Pharmacol. 356, 267–282. 10.1007/pl00005051 9303562

[B20] Lovinger D. M. White G. Weight F. F. (1989). Ethanol inhibits NMDA-activated ion current in hippocampal neurons. Science 243, 1721–1724. 10.1126/science.2467382 2467382

[B21] Ludvig N. Altura B. T. Fox S. E. Altura B. M. (1995). The suppressant effect of ethanol, delivered *via* intrahippocampal microdialysis, on the firing of local pyramidal cells in freely behaving rats. Alcohol 12, 417–421. 10.1016/0741-8329(95)00012-g 8519436

[B22] Martin D. Swartzwelder H. S. (1992). Ethanol inhibits release of excitatory amino acids from slices of hippocampal area CA1. Eur. J. Pharmacol. 219, 469–472. 10.1016/0014-2999(92)90491-l 1358646

[B23] Matthews D. B. Simson P. E. Best P. J. (1996). Ethanol alters spatial processing of hippocampal place cells: a mechanism for impaired navigation when intoxicated. Alcohol. Clin. Exp. Res. 20, 404–407. 10.1111/j.1530-0277.1996.tb01660.x 8730237

[B24] Okada S. Igata H. Sasaki T. Ikegaya Y. (2017). Spatial representation of hippocampal place cells in a T-maze with an aversive stimulation. Front. Neural Circuits 11:101. 10.3389/fncir.2017.00101 29321727 PMC5732186

[B25] O’Keefe J. Dostrovsky J. (1971). The hippocampus as a spatial map. Preliminary evidence from unit activity in the freely-moving rat. Brain Res. 34, 171–175. 10.1016/0006-8993(71)90358-1 5124915

[B26] O’Neill J. Senior T. J. Allen K. Huxter J. R. Csicsvari J. (2008). Reactivation of experience-dependent cell assembly patterns in the hippocampus. Nat. Neurosci. 11, 209–215. 10.1038/nn2037 18193040

[B27] Ryabinin A. E. (1998). Role of hippocampus in alcohol-induced memory impairment: implications from behavioral and immediate early gene studies. Psychopharmacology 139, 34–43. 10.1007/s002130050687 9768540

[B311] Schmitzer-Torbert N. Jackson J. Henze D. Harris K. (2005). Quantitative measures of cluster quality for use in extracellular recordings. Neurosci. 131, 1–11. 10.1016/j.neuroscience.2004.09.066 15680687

[B28] Shimizu K. Matsubara K. Uezono T. Kimura K. Shiono H. (1998). Reduced dorsal hippocampal glutamate release significantly correlates with the spatial memory deficits produced by benzodiazepines and ethanol. Neuroscience 83, 701–706. 10.1016/s0306-4522(97)00339-4 9483554

[B29] Singer A. C. Frank L. M. (2009). Rewarded outcomes enhance reactivation of experience in the hippocampus. Neuron 64, 910–921. 10.1016/j.neuron.2009.11.016 20064396 PMC2807414

[B30] Suh J. Foster D. J. Davoudi H. Wilson M. A. Tonegawa S. (2013). Impaired hippocampal ripple-associated replay in a mouse model of schizophrenia. Neuron 80, 484–493. 10.1016/j.neuron.2013.09.014 24139046 PMC3871857

[B31] Tokunaga S. McDaniel J. R. Morrow A. L. Matthews D. B. (2003). Effect of acute ethanol administration and acute allopregnanolone administration on spontaneous hippocampal pyramidal cell neural activity. Brain Res. 967, 273–280. 10.1016/s0006-8993(02)04266-x 12650988

[B32] White A. M. Best P. J. (2000). Effects of ethanol on hippocampal place-cell and interneuron activity. Brain Res. 876, 154–165. 10.1016/s0006-8993(00)02629-9 10973604

[B33] White A. M. Matthews D. B. Best P. J. (2000). Ethanol, memory and hippocampal function: a review of recent findings. Hippocampus 10, 88–93. 10.1002/(SICI)1098-1063(2000)10:1<88::AID-HIPO10>3.0.CO;2-L 10706220

[B34] Wilson M. A. McNaughton B. L. (1994). Reactivation of hippocampal ensemble memories during sleep. Science 265, 676–679. 10.1126/science.8036517 8036517

[B35] Yagi S. Igata H. Shikano Y. Aoki Y. Sasaki T. Ikegaya Y. (2018). Time-varying synchronous cell ensembles during consummatory periods correlate with variable numbers of place cell spikes. Hippocampus 28, 471–483. 10.1002/hipo.22846 29633414

